# Effects of Combining Music Therapy, Light Therapy, and Chromotherapy in the Treatment of Chronic Pain Patients: A Pilot Study

**DOI:** 10.1155/2024/3006352

**Published:** 2024-03-06

**Authors:** Alcira Suarez, Yannick Delgado, Alain Servais, Nicolas Verardi, Delphine Durand, Severine Litaneur, Vincent Wyart, Julien Nizard, Jean-Paul Nguyen

**Affiliations:** ^1^Pain Assessment and Treatment Center (CETD), Clinique Bretéché, Elsan Group, Nantes 44000, France; ^2^Société DYCOM SAS, Saint-Herblain 44800, France; ^3^Santé Atlantique, Elsan Group, Nantes, France; ^4^CETD CHU (University Hospital) Laennec, Nantes, France

## Abstract

**Background:**

It is currently considered that around 30% of chronic pain patients are totally refractory to medical treatment. Among patients who remain responsive to medical treatment, it is estimated that between 20% and 50% are likely to discontinue treatment due to severe side effects. Given these therapeutic difficulties, a significant number of patients turn to complementary therapies.

**Objective:**

The LineQuartz® is a medical device that combines 3 complementary therapies, namely, music therapy, light therapy, and chromotherapy. We propose to evaluate its effectiveness in chronic pain patients.

**Methods:**

Between October 2021 and October 2022, 44 patients aged between 23 and 85 years (mean: 55.4 years) were included in a prospective study. All patients had background pain intensity greater than 4/10 on the Numerical Pain Scale (NS). Treatment consisted of 4 half-hour sessions, divided into one session per week for 3 weeks (21 days). Patients were assessed by the Brief Pain Inventory (BPI) and the Hospital Anxiety and Depression scale (HAD) the day before starting treatment (Day 0) and the day after the end of treatment (Day 22).

**Results:**

Apart from the BPI item, “relationship with others,” all items improved significantly (*p* < 0.050). Background pain intensity (NS) and frequency of painful attacks improved very significantly (*p* < 0.001). The HAD anxiety subscore was also significantly improved (*p* < 0.001). *Discussion*. This open pilot study supports the idea that LineQuartz® has a place among complementary therapies dedicated to the treatment of chronic pain. However, these results need to be confirmed by a controlled study.

## 1. Introduction

Chronic pain is common, affecting over 30% of the French population [1]. It mainly concerns neuropathic pain, spinal pain, or diffuse pain. It is difficult to treat, rapidly affects quality of life, leads to a significant number of work stoppages, and is a priority in public health policy [2, 3].

Level 1 and 2 analgesics are recommended as 1st and 2^nd^ line treatments but often prove ineffective. Level 3 analgesics, mainly morphine, also often fail, and it is currently considered that around 30% of chronic pain patients are totally refractory to medical treatment [4]. Among patients who remain responsive to treatment, it is estimated that between 20% and 50% are likely to discontinue treatment due to severe side effects such as drowsiness, mood disorders, or cognitive impairment [5]. Given these therapeutic difficulties, a significant number of patients turn to alternative therapies [6] represented by complementary therapies [7, 8].

These therapies have been classified into 3 categories as follows [9–11]:

1. Therapies that involve physical manipulation through touch or exercise: acupressure, massage, chiropractic medicine, reflexology, osteopathy, Qi Gong, Tai Chi, Alexander technique, and yoga.
2. Body energy therapies: acupuncture, aromatherapy, homeopathy, light therapy, reiki, chromotherapy, polarity therapy, and therapeutic touch.
3. Mind body therapies: art therapy, visualization or guided imagery, hypnosis, meditation, relaxation, biofeedback, dance or movement therapy, and music therapy.

These different therapies contribute to a global approach to pain, in association with the multimodal approach (including psychological, social, and professional care) and therapies aimed at treating the pain itself (analgesic drugs and/or central and peripheral nervous system stimulation techniques). They share the following certain common features [9]:

- They work in harmony with the body's self-healing mechanisms.
- They are “holistic,” i.e., they treat the whole person.
- They encourage patients to take an active part in the process.
- They focus on well-being and disease prevention.

The LineQuartz® is a medical device that combines 3 complementary therapies, namely, music therapy, light therapy, and chromotherapy.

Music therapy, especially in its receptive form, is recognized as being able to modify pain sensation [12–14]. Light therapy acts on the body clock and circadian rhythm. It has been shown to act on sleep disorders [15] and mood disorders [16, 17]. Chromotherapy uses colors to modulate or produce physiological effects. The most studied effect is on anxiety and stress, often encountered in chronic pain patients [18].

The aim of this article is to evaluate the efficacy of LineQuartz® in the treatment of chronic pain patients. This is a pilot study which will serve to lay the foundations for a controlled trial.

## 2. Methods

### 2.1. Inclusion Criteria

The inclusion criteria included the following: patients aged at least 18 years, chronic pain evolving for at least 3 months, and pain not soothed by level 1 and 2 analgesics and intensity assessed as at least 4/10 on the Numerical Pain Scale (NS).

### 2.2. Exclusion Criteria

The exclusion criteria included the following: patient unable to be properly assessed (dementia and psychosis), patient refusing to sign the information and informed consent document, withdrawal of consent, appearance of acute pain during the study period, chronic conditions likely to interfere with the effectiveness of the planned measures (visual or hearing problems), and introduction during the study of other analgesic therapeutic strategies (e.g., acupuncture), which may interfere with the results.

### 2.3. Study Design

The study was an open pilot study, with a case series design, carried out in 44 patients included between October 2021 and October 2022.

Patients were recruited from 10 therapists located in France (*n* = 8) or Belgium (*n* = 2). All therapists had a recognized medical or paramedical activity, i.e., physiotherapists (*n* = 3), occupational therapists (*n* = 2), nurse (*n* = 2), osteopaths (*n* = 1), dentist (*n* = 1), or neurophysiologist (*n* = 1). The detailed educational content of the LineQuartz® training course is available at https://www.linequartz.com (request permission to view this chapter at direction@dycomsas.com). The principles of evidence-based medicine [19, 20] and ethics [21] are integrated into all levels of this training.

All patients signed an informed consent form. The protocol has been approved by the Nantes Hospital Ethics Committee (GNEDS (Groupe Nantais d'Éthique dans le Domaine de la Santé)), with the reference 23-101-08-280.

**Figure 1:**
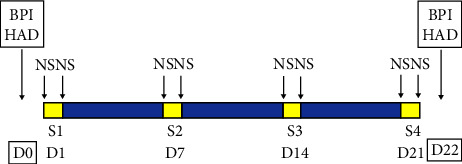
Protocol schedule. The BPI (Brief Pain Inventory) and the HAD (Hospital Anxiety and Depression scale) were assessed before the start (D0) and after the end (D22) of the therapeutic protocol. The protocol consisted of 4 therapeutic sessions (S1–S4), each lasting 30 minutes and spaced 7 days apart (D1–D7, D7–D14, and D14–D21). The Numeric Pain Rating Scale (NS) was collected just before and just after each session.

All patients were assessed before treatment (Day 0 (D0)) and after the protocol (Day 22 (D22)) using the following scales (Figure 1).

(1) Numerical Pain Scale (NS): the patient rated his or her background pain intensity with a number between 0 (no pain) and 10 (maximum imaginable pain). For overall assessment of the procedure (at D0 and D22), we used the average pain level recorded over the last 24 hours (item 5 of the Brief Pain Inventory (BPI) pain assessment) [22]. To assess the effectiveness of the 4 therapeutic sessions, we used the NS value recorded just before and just after the session (BPI item 6).
It is accepted that the cutoff point between mild and moderate pain (affecting activities of daily living) is 4/10 on the Numerical Pain Scale [23]. In many therapeutic trials, patients presenting with pain ≤4/10 are excluded from the study, as they do not present pain judged to be sufficiently intense [24]. For these reasons, we chose to include only patients with pain ≥4/10.
(2) Number of painful attacks per day (if any).
(3) Brief Pain Inventory interference items [22]: a numerical scale ranging from 0 to 10 was used to assess several items generally affected by chronic pain.
  - General activity
  - Mood
  - Walking ability
  - Normal work
  - Relationships with others
  - Sleep
  - Enjoyment of life
(4) Hospital Anxiety and Depression scale (HAD) [25]: anxiety and depression were assessed separately by 7 questions, resulting in a score ranging from 0 to 21. The total score is the sum of the 2 scores, which can range from 0 to 42.
(5) Each patient was asked at D22 whether they had been satisfied with the treatment and whether they would recommend it to someone else suffering from chronic pain.

Prior to treatment, the DN4 questionnaire [26] was analyzed to assess the importance of the neuropathic component of the pain.

Medical analgesic treatment was recorded in the patient's file at D0 and D22 for an evaluation using the Medication Quantification Scale (MQS version 1992) [27, 28]. Each drug has a score that depends on the potential severity of side effects (detriment) (e.g., score of 2 for nonsteroidal anti-inflammatory drugs and score of 6 for strong opioid derivatives) and daily doses (subtherapeutic = 1, minimal therapeutic = 2, maximal = 3 and supratherapeutic = 4). For example, a patient with an MQS score between 2 and 4 is on “weak” treatment and above 6 as on “strong” treatment.

Many potential candidates for inclusion in this protocol lived far from the therapists. It was deemed difficult to get them all to return for an evaluation that would be desirable 15–30 days after the end of treatment. For this pilot study, we only scheduled a final assessment the day after the end of treatment (D22).

The LineQuartz® is the device that delivers the treatment to be assessed. It combines 3 complementary therapies in a single device, namely, music therapy, light therapy, and chromotherapy. The protocol consists of one session per week for 3 weeks (S1 (D1), S2 (D7), S3 (D14), and S4 (D21)). Each session lasts 30 minutes.

### 2.4. Therapeutic Procedure

**Figure 2:**
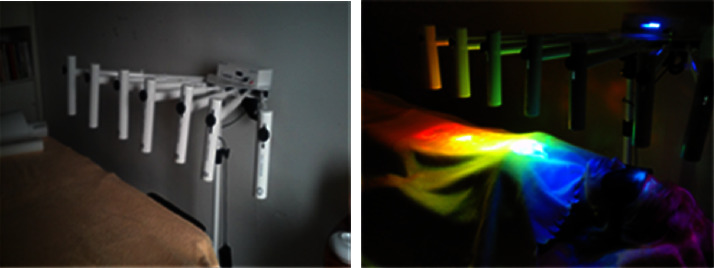
The LineQuartz® light and color therapy system. CE-certified lamps comply with European standards for medical devices that emit neither ultraviolet nor infrared light.

The device has 7 telescopic tubes that can be oriented above the person to be treated (Figure 2). The ends of the 7 telescopic tubes are fitted with height and side adjustable tubes. Each tube is fitted with a specific quartz crystal oriented on the patient's vertical axis, approximately 30 centimeters from his or her body. Each tube has a specific color filter, in line with the principle of chromotherapy. The device was patented in December 2013 by engineer Mr Yannick Delgado and built by Societé DYCOM SAS (Saint Herblain 44800 France). It was recognized as a medical device (under European Directive 93/42/EEC according to Annex IX relating to medical devices. Article II Classification: Class I DM noninvasive device) on January 15, 2016.

Each of the 4 sessions has a specific objective. During the session, the patient lies supine, covered by a white sheet (Figure 2). Headphones are placed over the ears.

  1st session: the aim is to provide the body with precise luminous and vibratory information through the diffusion of electromagnetic light waves (less than 90 lumens) combined with the diffusion of musical vibrations set at a frequency of 432 Hz. This type of session should induce frequency homeostasis (see discussion). Other sessions differ in the choice of themes used in music therapy.
  2nd session: the aim is to act on 2 components involved in biorhythms (emotional and intellectual) by broadcasting musical works by 2 composers. (1) A romantic work by Schubert, with the aim of stimulating the emotional cortical and subcortical areas known to be activated when listening to music. (2) A work by Bach, with the aim of stimulating the more analytical areas of listening to well-known pieces.
  Session 3: listening to a well-known work by Mozart, with the aim of stimulating cortical areas linked to memory, proprioception, and spatial perception.
  Session 4: sacred music by composer Michel Pepe promotes relaxation and pain reduction by distending time and space.

### 2.5. Statistical Analysis

The difference between the main variables recorded before and after treatment was assessed by analysis of variance (ANOVA) and the nonparametric Mann–Whitney test.

Effect size was assessed using Jacob Cohen's d-index [29]. A value of 0.2 corresponds to a weak effect, 0.5 to a medium effect, 0.8 to a strong effect, and 1.3 to a very strong effect.

## 3. Results

The 32 women and 12 men ranged in age from 23 to 85 years (mean: 55.4 years ± 12.7). All patients had background chronic pain intensity greater than 4/10 on the Numerical Pain Scale (NS). Pain duration ranged from 6 months to 50 years (mean: 9.5 years ± 11.3). Causes of pain were classified into 8 categories:

1. Rheumatic diseases (*n* = 11),
2. Diffuse polyalgic syndromes (*n* = 7): diffuse pain not meeting the diagnostic criteria for fibromyalgia (ACR 2010 criteria [30]).
3. Spinal pain (*n* = 6),
4. Fibromyalgia (*n* = 5): ACR 2010 criteria,
5. Neuralgia (*n* = 4),
6. Headache (*n* = 3),
7. Algodystrophy (*n* = 2).
8. Other (*n* = 6): Ehlers–Danlos disease (*n* = 1), Berger's disease (*n* = 1), endometriosis (*n* = 1), cystitis (*n* = 1), pelvic pain (*n* = 1), and small-fiber neuropathy (*n* = 1).

For the analysis of results, “diffuse polyalgic syndromes” and “fibromyalgia” were grouped together under the term “widespread pain” (*n* = 12).

Nineteen patients were not taking any medication for pain relief. Thirteen had treatment considered “weak,” with an MQS score between 2 and 4 (mean: 3 ± 0.5 (standard deviation)). We combined these 2 groups into a single group (group 1) with a mean MQS score of 1.2 ± 1.5. Twelve patients had treatment considered as “strong” (group 2), with an MQS score between 6 and 18 (mean: 10.6 ± 3.8). There was a significant difference in the MQS score between group 1 and group 2 (*p* < 0.001).

Patients in group 1 had a mean pretreatment NS score of 5.7 ± 1.4 and those in group 2 a score of 6.5 ± 0.9. The difference was marginally significant (*p*=0.042).

The NS score of patients in group 1 improved on average by 52.6% (±27.4) and that of patients in group 2 by 39.8% (±29.9). The difference was not significant (*p*=0.396).

There was no change in analgesic treatment between D0 and D22 (see discussion).

The results (Table 1), assessed at D22 (end of treatment), show a significant improvement (*p* < 0.050) in the following variables (in order of significance (p) and effect size (d)):

  Background pain (*p* < 0.001 and *d* = 1.7)
  Number of painful attacks per day (*p* < 0.001 and *d* = 1.7)
  Anxiety (*p* < 0.001 and *d* = 0.8)
  General activity (*p* < 0.001 and *d* = 0.8)
  Sleep (*p* < 0.001 and *d* = 0.7)
  Normal work (*p* < 0.001 and *d* = 0.7)
  Mood (*p* < 0.002 and *d* = 0.6)
  Walking ability (*p* < 0.004 and *d* = 0.6)
  Depression (*p* < 0.008 and *d* = 0.5)
  Enjoyment of life (*p*=0.010 and *d* = 0.5)

The effect size (d) was found to be large (*d* ≥ 0.8) or very large (*d* ≥ 1.3).

Improvement was less or not significant on items concerning depression (*p* < 0.008 and *d* = 0.5), enjoyment of life (*p*=0.010 and *d* = 0.5), and relationships with others (*p*=0.055 and *d* = 0.4).

Most patients (38/44 (86.4%)) were satisfied with the treatment, and 88.6% of them (39/44) would recommend the same treatment to someone with the same chronic pain.

No side effects have been reported.

Each therapy session brought an improvement in the NS (on average, 1.8 point/10), which was significant for the first 3 sessions (Table 2). In the days following the session, the NS worsened by an average of 1.2 point per week. Looking ahead, it could be estimated that after a treatment of just 4 therapeutic sessions, patients can lose an average of 3.6 points (1.2 × 3) 3 weeks after stopping treatment, bringing patients' pain levels back to their initial level (2.8 + 3.6 = 6.4).

If we consider the number of significantly improved items, spinal pain (*n* = 10) comes top with the following 5 improved items: background pain, number of painful attacks, general activity, normal work, and anxiety. In 2^nd^ position comes widespread pain (*n* = 12) with the following 4 improved items: background pain, number of painful attacks, sleep, and anxiety. Rheumatic pain (*n* = 11) comes in 3^rd^ with only the following 3 items improved: background pain, number of painful attacks, and general activity (Table 3).

The length of time the pain had been present before treatment seems to influence the outcome. When pain duration was relatively short (between 6 months and 2 years), there was a significant improvement in the following 7 items: background pain, number of painful attacks, general activity, mood, normal work, sleep, and anxiety. When pain lasted between 2 and 8 years, the improvement concerned the following 6 items: background pain, number of painful attacks, general activity, enjoyment of life, anxiety, and depression. When the pain lasted for many years (between 8 and 50 years), the improvement concerned only the following 4 items: background pain, number of painful attacks, general activity, and walking ability (Table 4).

Analysis of the DN4 questionnaire enabled us to identify 27 patients with a score ≥4 and therefore presenting a predominant or exclusively neuropathic component or pain. These were widespread pain (*n* = 10), joint pain (rheumatic diseases) (*n* = 6), spinal pain (*n* = 4), or neuralgic pain (*n* = 3). Four other patients had pain associated with endometriosis, algodystrophy, small-fiber neuropathy, and headache.  Table 5 compares the results of patients with neuropathic pain with those without a neuropathic component (*n* = 17). These results suggest that treatment is more effective in the neuropathic pain group, with 8 items statistically significantly improved (*p* < 0.050), 5 of which were highly significant (*p* < 0.003), i.e., background pain, number of painful attacks, general activity, sleep, and anxiety (Table 5).

## 4. Discussion

This study suggests that LineQuartz® can improve chronic pain patients in almost all BPI-assessed symptoms. The most improved symptoms were background pain and the number of painful attacks. Continuous background pain is more common in widespread pain. Painful attacks are often “mechanical,” triggered by movement or posture, and are more often found in spinal pain. Perhaps, this is why these 2 pathologies seemed to respond best to treatment.

It should be noted that the treatment proved highly effective, above all, in the areas where we might expect it to be, given the supposed mechanisms of action of the therapies implemented, i.e., pain (music therapy), sleep (light therapy), mood (light therapy), and stress (chromotherapy). However, the treatment also proved effective in areas that suggest an improvement in “motor and/or motivational” abilities, such as improving general activity and work capacity.

It is likely that the follow-up was too short (22 days) to allow proper assessment of depression, enjoyment of life, and relationships with other items and observing a change in medical analgesic treatment. Given the potential duration of the treatment effect (around 3 weeks), assessment should also be carried out at D37, D43, and D52. The relatively modest effect size for these 3 items (between 0.41 and 0.54) suggests that a study involving a larger number of cases might have yielded a more significant result.

The effect of music therapy on pain is widely appreciated. In a study of cancer patients, it was shown that a receptive music therapy session lasting 20–25 minutes provided an average improvement of 58% on the visual analog pain scale and that this effect lasted about 30 minutes after the end of the session [31]. However, patients questioned on the contribution of music therapy highlighted the following 4 items that did not directly concern pain relief: (1) forgetting one's illness, total disconnection, (2) relaxation, comfort in relation to care, (3) finding oneself again, opening to the psyche, directing one's fear or emotions, and transforming them, and (4) good mood, positive thinking, dynamism, and morale. The authors have thus considered music therapy to be a form of supportive care. In an analysis of the value of music therapy in oncology, music therapy was considered most useful for improving patients' moods [32], while complementary therapies likely to improve pain were represented more by massage therapy, relaxation techniques, and acupuncture [7]. However, the direct effect of music therapy on pain intensity has been demonstrated. In a controlled study of cancer patients, after a 30-minute session of receptive music therapy, patients in the treatment group (*n* = 62) improved significantly in pain intensity (*p* < 0.001) compared with a control group (*n* = 64) [33]. The mechanism of action of music therapy was investigated by analyzing the changes induced in functional imaging by listening to different types of musical pieces. In functional MRI, significant activation of the nucleus accumbens (NAc) and ventral tegmental area (VTA) was found [34]. These structures are part of the limbic system and contain dopaminergic neuronal circuits involved in pain modulation and reward phenomena [35]. Pain modulation would involve connections between the NAc, the prefrontal cortex, and noradrenergic neural circuits involving neurons in the locus coeruleus (LC) [36]. Activity in the prefrontal cortex is thought to depend in part on interactions between dopaminergic and noradrenergic neurons [36, 37]. The prefrontal cortex is itself connected to the anterior cingulate cortex, insula, amygdala, and hippocampus, which are structures involved in modulating the affective component of pain [38]. The role of NAc in modulating neuropathic pain and its ability to prevent a transition to chronicity has been demonstrated experimentally [39]. Perhaps, this is why LineQuartz® has proved particularly effective on neuropathic pain and pain that has been evolving for less than 2 years. The action of music therapy on dopaminergic circuits that modulate prefrontal cortex activity could be enhanced by also acting on noradrenergic circuits that also modulate prefrontal cortex activity, as can-do occipital nerve stimulation (ON) by tDCS (transcranial direct current electrical stimulation) [40]. Another mechanism for the action of music therapy involves the characteristics of sound, in particular its different vibratory frequencies [41]. Normally, in a state of homeostasis, the electromagnetic fields emanating from the body and its various organs and tissues have a certain vibratory frequency. If this frequency changes in a certain area of the body, that area will no longer be in resonance with the frequencies of the other parts of the body and may malfunction. Certain frequencies, such as 432 Hz, are supposed to re-establish an adequate resonance frequency. This can be called frequency homeostasis.

Light therapy essentially acts on circadian rhythms via melatonin secretion by the epiphysis, influenced by the light/dark cycle [42, 43]. It is easy to see how light therapy could improve sleep disorders and seasonal depression, a priori linked to a relative lack of light in winter [44]. Improving sleep disorders and mood are the main actions of light therapy [45]. It can thus indirectly improve the quality of life of chronic pain patients. However, the visual or light signal picked up by the retina is transmitted to the epiphysis via a noradrenergic pathway that passes through the brainstem before reaching the superior cervical ganglion and then the epiphysis [42]. Light stimulation of noradrenergic pathways passing through the brainstem [45] could potentiate the effect of music therapy, which also indirectly depends on noradrenergic pathways linking the LC to the prefrontal cortex [32]. This potentiating effect could possibly explain why LineQuartz® could have a greater effect than music therapy alone.

In LineQuartz®, chromotherapy is not specifically aimed at treating pain, but is placed in a more global context of repairing an energetic imbalance that may be caused by stress. This imbalance may have consequences for the functioning of neural circuits involved in pain [46]. Chromotherapy generates different types of waves, notably electromagnetic, which are supposed to act on the hormonal system but also at cellular level, helping to maintain an energetic balance that promotes harmonious functioning of the organism. This principle is like that of REAC (Radio Electric Asymmetric Conveyer) technology [47, 48], which uses a burst stimulation current (250 ms and 5.8 GHz) applied to the auricle or to a specific area to be treated. The objective efficacy of this technique is reflected in an improvement in maladaptive motor behavior, which is thought to be the result of repeated stress episodes encountered throughout life. This behavior is assessed by the “functional dysmetria assessment.” Very interesting results, concerning physical activity and motor behavior have been obtained in patients with neurodegenerative diseases [49, 50]. It is perhaps this same mechanism that explains the improvement in general activity and work capacity found in our patients.

The main limitations of this study are the absence of a control group. The results of this study should, therefore, be considered as preliminary and should be confirmed by a controlled study including an arm without LineQuartz treatment. The lack of follow-up is also a limitation of this study. A future study should include a follow-up of at least 1 month after the end of treatment. The recruitment of patients by therapists rather than primary care providers may introduce bias into the sample. In the next study, patients will be recruited from two pain centers in Nantes.

## 5. Conclusion

This study supports the idea that LineQuartz® has a place among complementary therapies dedicated to the treatment of chronic pain. The data from this study encourage further clinical investigation into the use of LineQuartz in the treatment of chronic pain patients. Music therapy's mechanism of action at the level of dopaminergic circuits that modulate prefrontal cortex activity could be enhanced by tDCS stimulation of occipital nerves (tDCS-ON) [36]. A controlled study including a control arm, an arm treated only with LineQuartz®, and an arm including a tDCS-ON session performed at the same time as LineQuartz® treatment would be very interesting. This protocol should include an evaluation performed at least 1 month after the end of treatment and include an assessment of functional dysmetria. The best candidates for inclusion in this protocol should be patients suffering from neuropathic pain for less than 2 years.

## Figures and Tables

**Table 1:** Evolution of BPI and HAD scores collected before (D0) and after treatment (D22) (see BPI item details in text).

	Pretreatment		Posttreatment		Change	% improvement	*p*	*d*
	Mean	SD	Mean	SD				
Background pain/10^*∗*^	6.0	1.4	3.0	1.7	3.0	49	<0.001	1.7
Painful attacks (*n*/day)	7.7	1.8	4.5	1.8	3.2	39	<0.001	1.7
General activity/10	6.1	2.7	3.9	2.5	2.2	35	<0.001	0.8
Mood/10	5.4	2.9	3.6	2.5	1.8	29	<0.002	0.6
Walking ability/10	4.8	2.8	3.0	2.6	1.8	32	<0.004	0.6
Normal work/10	6.5	2.1	4.5	2.6	2.0	30	<0.001	0.8
Relation with other people/10	4.3	2.9	3.1	2.7	1.2	15	=0.055	0.4
Sleep/10	5.4	2.9	3.1	2.6	2.3	40	<0.001	0.8
Enjoyment of life/10	4.4	3.3	2.6	2.6	1.8	35	=0.010	0.5
Anxiety/21	11.1	4.1	7.4	3.7	3.7	30	<0.001	0.9
Depression/21	7.8	4.3	5.5	3.9	2.3	26	<0.008	0.5

Apart from the “relationships with others” item, all other BPI items and the 2 HAD items improved significantly (*p* < 0.050). The effect size was very large on background pain (*d* = 1.7) and number of painful attacks per day (*d* = 1.7) items. ^*∗*^: Numerical scale corresponding to average pain over the last 24 hours (BPI item 5). SD: standard deviation. *n*/day: number of painful attacks per day.

**Table 2:** Evolution of pain intensity (numerical pain scale ranging from 0 to 10) before and after each therapeutic session.

	Pretreatment		Posttreatment		Change (S)	Change (IS)	*p*	*d*
	Mean	SD	Mean	SD				
Session 1:/10^*∗∗*^	6.4	1.7	4.4	1.9	−2		<0.001	1.0
Session 2:/10^*∗∗*^	5.2	2.0	3.3	1.6	−1.9	0.8	<0.001	0.9
Session 3:/10^*∗∗*^	4.9	2.6	3.1	2.2	−1.8	1.6	<0.001	0.7
Session 4:/10^*∗∗*^	4.3	2.5	2.8	2.4	−1.5	1.2	<0.007	0.6
Mean					−1.8	1.2		

^*∗∗*^Numerical Pain Scale (NS) recorded just before and just after each session (S). IS: change recorded between the end of one session and just before the next. After one week, the sessions lose their effectiveness, with an average loss of 1.2 point on the NS.

**Table 3:** Evolution of BPI and HAD scores between D0 and D22 on according to the type of pathology responsible for the pain.

	Pretreatment		Posttreatment		Change	% improvement	*p*	*d*
	Mean	SD	Mean	SD				
Rheumatic pain								
Background pain/10	5.9	1.1	2.5	1.8	3.4	56	<0.001	1.9
Painful attacks (*n*/day)	7.6	1.2	5.0	1.7	2.6	35	<0.001	2.2
General activity/10	5.5	2.8	3.3	2.1	2.2	41	=0.016	0.8
Mood/10	3.7	2.8	2.6	2.0	1.1	28	=0.323	0.4
Walking ability/10	4.8	1.9	3.5	2.3	1.3	32	=0.144	0.7
Normal work/10	4.8	1.9	3.5	2.3	1.3	31	=0.144	0.7
Relation with other people/10	2.9	2.8	2.1	2.5	0.8	37	=0.521	0.3
Sleep/10	3.5	2.8	2.5	2.4	1.0	35	=0.349	0.4
Enjoyment of life/10	2.8	2.9	1.9	2.2	0.9	35	=0.499	0.3
Anxiety/21	8.1	5.0	6.4	4.9	1.7	23	=0.476	0.3
Depression/21	6.4	3.9	4.6	4.2	1.8	37	=0.274	0.5
Widespread pain								
Background pain/10	5.8	1.7	2.4	1.4	3.4	57	<0.001	2.5
Painful attacks (*n*/day)	7.3	2.7	3.9	2.0	3.3	42	<0.001	1.2
General activity/10	6.5	2.5	4.5	3.2	2.0	31	=0.077	0.8
Mood/10	5.3	3.2	3.8	2.7	1.5	20	=0.267	0.4
Walking ability/10	5.1	2.7	3.5	3.0	1.6	27	=0.128	0.6
Normal work/10	6.8	2.0	5.3	2.6	1.5	20	=0.142	0.6
Relation with other people/10	4.4	3.1	3.2	2.9	1.2	−11	=0.351	0.4
Sleep/10	6.8	2.7	3.7	2.5	3.1	37	=0.012	1.2
Enjoyment of life/10	5.2	3.7	3.3	3.0	1.9	27	=0.219	0.5
Anxiety/21	12.7	3.7	7.9	2.6	4.8	32	<0.004	1.3
Depression/21	8.5	5.6	4.8	3.5	3.7	33	=0.060	0.7
Spinal pain								
Background pain/10	5.9	1.2	3.9	2.0	2.0	35	=0.012	1.0
Painful attacks (n/day)	8.2	1.1	5.6	1.8	2.6	32	<0.002	2.3
General activity/10	5.7	3.2	3.5	2.5	2.2	37	=0.021	0.7
Mood/10	5.9	2.8	4.0	2.2	1.9	27	=0.051	0.7
Walking ability/10	5.6	3.4	3.7	2.8	1.9	24	=0.118	0.6
Normal work/10	7.5	1.6	5.7	1.6	1.8	23	=0.025	1.2
Relation with other people/10	5.0	3.0	4.2	2.6	0.8	14	=0.376	0.3
Sleep/10	5.7	2.6	4.2	2.7	1.5	21	=0.164	0.6
Enjoyment of life/10	5.2	3.3	2.7	2.6	2.5	47	=0.065	0.8
Anxiety/21	10.6	2.5	7.8	4.1	2.8	28	<0.002	1.1
Depression/21	6.4	4.4	6.1	4.7	0.3	1	=0.896	0.1

In patients with chronic spinal pain, 5 items improved, with 4 of them showing a significant effect size (between 1.0 and 2.2).

**Table 4:** Evolution of BPI and HAD scores between D0 and D22 according to duration of pain evolution.

	Pretreatment		Posttreatment		Change	% improvement	*p*	*d*
	Mean	SD	Mean	SD				
6 months--> 2 years (*n* = 16)								
Background pain/10	6.3	1.3	3.6	1.7	2.7	41	<0.001	1.6
Painful attacks (*n*/day)	8.1	1.2	5.1	2.0	3.0	37	<0.001	2.5
General activity/10	6.1	2.5	4.0	1.8	2.1	33	<0.001	0.8
Mood/10	6.3	2.9	3.5	2.5	2.8	41	<0.005	1.0
Normal work/10	7.0	1.9	4.3	2.8	2.7	34	<0.004	1.0
Sleep/10	6.2	2.8	2.4	2.5	3.8	59	<0.001	1.4
Anxiety/21	10.1	4.0	7.1	4.7	3	30	=0.043	0.8
3--> 8 years (*n* = 14)								
Background pain/10	5.7	1.2	2.5	1.6	3.2	56	<0.001	2.0
Painful attacks (n/day)	7.0	2.5	3.5	1.9	3.5	47	<0.001	1.4
General activity/10	6.3	2.2	4.1	3.0	2.2	36	=0.030	1.0
Enjoyment of life/10	5.0	3.1	2.7	2.4	2.3	37	=0.035	0.7
Anxiety/21	13.1	3.8	7.9	2.5	5.2	36	<0.001	1.4
Depression/21	8.6	3.8	6.3	3.4	2.3	19	=0.037	0.6
9--> 50 years (*n* = 14)								
Background pain/10	5.9	2.9	2.9	1.7	3.0	52	<0.001	1.8
Painful attacks (n/day)	8.1	1.3	5.3	1.0	2.8	34	<0.001	2.2
General activity/10	5.9	3.4	3.6	2.6	2.3	37	=0.015	0.7
Walking ability/10	5.3	2.1	3.5	2.9	1.8	30	=0.032	0.4

Only scores that improved significantly (*p* < 0.050) are shown. When pain lasted less than 2 years, the improvement concerned 7 items. The improvement was highly significant for background pain, number of painful attacks, general activity, and sleep.

**Table 5:** Evolution of BPI and HAD scores between D0 and D22 according to the presence or absence of neuropathic pain.

	Pretreatment		Posttreatment		Change	% improvement	*p*	*d*
	Mean	SD	Mean	SD				
Neuropathic pain								
Background pain/10	6.2	1.4	3.1	1.7	3.1	49	<0.001	1.8
Painful attacks (*n*/day)	7.8	2.1	4.6	2.0	3.2	40	<0.001	1.5
General activity/10	6.9	1.8	4.4	2.4	2.5	36	<0.001	1.0
Mood/10	5.9	2.7	4.1	2.4	1.8	25	=0.018	0.7
Normal work/10	6.7	2.0	4.9	2.6	1.8	25	<0.006	0.7
Sleep/10	6.1	2.5	3.7	2.5	2.4	32	<0.002	1.0
Anxiety/21	11.6	3.6	8.0	3.4	3.6	28	<0.001	1.0
Depression/21	8.2	4.0	5.7	3.2	2.5	24	=0.014	0.6
Non neuropathic pain								
Background pain/10	5.6	1.3	2.9	1.8	2.7	50	<0.001	1.5
Painful attacks (*n*/day)	7.6	1.3	4.8	1.6	2.8	38	<0.001	1.8
Walking ability/10	5.3	2.8	2.8	2.3	2.5	47	=0.015	0.9
Normal work/10	6.1	2.3	3.8	2.4	2.3	38	=0.011	1.0
Anxiety/21	10.2	4.8	6.6	4.2	3.6	35	=0.011	0.8

Only scores that improved significantly (*p* < 0.050) are shown. These results suggest that treatment is more effective in the neuropathic pain group, with 8 items statistically significantly improved (*p* < 0.050), including the following 5 highly significantly (*p* < 0.003): background pain, number of painful attacks, general activity, sleep, and anxiety.

## Data Availability

The data used to support the findings of this study are available from the corresponding author upon request.
